# Draft Genome Sequences of 10 Multidrug-Resistant Escherichia coli Strains Isolated from Piglets in Peru

**DOI:** 10.1128/mra.00706-22

**Published:** 2022-09-26

**Authors:** Luis Alvarez, Denis Carhuaricra, Joel Palomino-Farfan, Sonia Calle, Lenin Maturrano, Juan Siuce

**Affiliations:** a Laboratorio de Microbiología y Parasitología, Sección de Bacteriología y Micología, Facultad de Medicina Veterinaria, Universidad Nacional Mayor de San Marcos, Lima, Perú; b Grupo de Investigación SANIGEN, Facultad de Medicina Veterinaria, Universidad Nacional Mayor de San Marcos, Lima, Perú; University of Arizona

## Abstract

Pathogenic Escherichia coli frequently causes diarrhea on pig farms. Here, we report the draft genome sequences of 10 strains of multidrug-resistant E. coli isolated from piglets.

## ANNOUNCEMENT

Pathogenic Escherichia coli is the most common cause of diarrhea in newborn and postweaning piglets around the world (1). In addition, high levels of multidrug resistance documented on animal farms in different countries is a serious threat for public health (2). Here, we report 10 sequenced E. coli strains, isolated from 10 piglets with clinical signs of diarrhea in Lima, Peru.

Isolates (*n* = 119) were recovered from intestinal samples of piglets 8 weeks of age or less with diarrhea and that had undergone necropsy for routine diagnosis. The gut samples were sent by veterinarians from pig farms to the Bacteriology Laboratory of the Faculty of Veterinary Medicine of the Universidad Nacional Mayor de San Marcos during 2020. A sample of intestine (1 cm^2^) was taken aseptically to directly inoculate the intestinal mucosa onto MacConkey agar (Merck, Germany) and streaked onto plates, which were then incubated at 37°C for 18 to 24 h. Identification of the isolated bacterial colonies was carried out by observing lactose fermentation in MacConkey agar (Merck). Confirmation was carried out by means of a panel of conventional biochemical tests (3). Antimicrobial susceptibility testing was performed on Mueller-Hinton agar (Merck) using the disk diffusion method, as described in the CLSI guidelines (4). The isolates were stored at −20°C in a cryoprotective medium.

For sequencing, the 10 isolates most resistant to antibiotics from different gut samples were selected. DNA was extracted from purified colonies grown in Luria-Bertani broth (Merck). DNA from each strain was extracted using the GeneJET genomic DNA purification kit (Thermo Fisher Scientific, USA) following the manufacturer’s instructions. The DNA quality and quantity were determined using a DS-11FX spectrophotometer (DeNovix Inc., USA) and a Qubit 2.0 fluorometer (Invitrogen, USA). Sequencing libraries were generated using the Nextera XT DNA library preparation kit (Illumina, San Diego, CA) according to the manufacturer’s protocol, and sequencing reactions were run on an Illumina MiSeq instrument in 2 × 250-bp paired-end format. Filtering of the low-quality sequence reads and *de novo* assembly of the high-quality sequences were performed using Trimmomatic v0.36 (5) and the SPAdes v3.13.0 genome assembler (6). QUAST v5.1 was employed to check the assembly quality and the completeness of the draft genomes and to compare contig statistics (7). Annotation of the assembled sequences was carried out using Prokka v1.14.5 (8). Antimicrobial resistance genes were annotated using ResFinder v4.1 (9, 10). Default parameters were used for all software tools.

### Data availability.

The data have been deposited at GenBank under BioProject accession number PRJNA839166. The GenBank, BioSample, and SRA accession numbers are provided in Table 1.

**TABLE 1:** Genomic features of 10 multidrug-resistant Escherichia coli strains from piglets

Strain	No. of reads	Total amt of DNA sequenced (bp)	GC content (%)	No. of contigs	*N*_50_ (bp)	Coverage (×)	No. of CDSs	GenBank accession no.	BioSample accession no.	SRA accession no.
EC204K	1,396,352	5,537,851	50.57	260	88,618	119.55	5,330	JAMJPG000000000	SAMN28512380	SRR21236072
EC206K	606,645	5,070,640	50.36	275	48,206	37.8	4,811	JAMJPF000000000	SAMN28512381	SRR21236071
EC225K	367,181	4,787,096	51.01	305	39,336	23.37	4,444	JAMJPE000000000	SAMN28512382	SRR21236070
EC237K	332,447	4,822,000	50.89	335	30,987	20.08	4,564	JAMJPD000000000	SAMN28512383	SRR21236069
EC247K	1,006,378	5,076,016	50.72	190	71,078	68.98	4,732	JAMJPC000000000	SAMN28512384	SRR21202685
EC248K	1,068,753	4,881,361	50.59	186	65,556	73.23	4,613	JAMJPB000000000	SAMN28512385	SRR21236068
EC254K	1,027,206	4,719,884	50.75	201	57,703	79.6	4,396	JAMJPA000000000	SAMN28512386	SRR21236067
EC258K	1,332,283	5,250,274	50.48	264	57,368	98.49	5,053	JAMJOZ000000000	SAMN28512387	SRR21236066
EC273K	1,083,163	5,500,467	50.36	390	48,645	71.6	5,340	JAMJOY000000000	SAMN28512388	SRR21236065
EC275K	2,037,400	5,539,502	50.27	320	64,175	129.39	5,398	JAMJOX000000000	SAMN28512389	SRR21236064
